# Assessment of Knowledge, Attitude and Practices (KAP) Towards Utilization of *Justicia heterocarpa* (Mwidu) in Kiroka Village, Morogoro Rural Tanzania

**DOI:** 10.1002/fsn3.71197

**Published:** 2025-11-12

**Authors:** Zenorina Aloyce Swai, Frida Albinusi Nyamete, Valerian C. K. Silayo

**Affiliations:** ^1^ Department of Food Science and Agro‐Processing School of Engineering and Technology Sokoine University of Agriculture Morogoro Tanzania

**Keywords:** food security, indigenous vegetable, socio‐demographic, traditional knowledge

## Abstract

This exploratory study examined the knowledge, attitudes, and practices concerning *Justicia heterocarpa,* a widely recognized indigenous leafy vegetable (ILV) within the Waluguru community in Kiroka Village, Morogoro Rural District Tanzania. The study's objective was to identify opportunities and challenges for the vegetable's enhanced application in the local context. A cross‐sectional survey was conducted among 174 households inKiroka Village, using a structured questionnaire and a snowball sampling technique. Data were analyzed using SPSS version 27.0. Descriptive statistics were used to summarize the socio‐demographic characteristics of the respondents and the distribution of KAP scores. Chi‐squared tests were employed to examine the associations between categorical socio‐demographic factors and categorical KAP levels. Pearson correlation coefficients were calculated to assess the linear relationships between continuous KAP scores and continuous socio‐demographic variables. Ordinal regression models were used to examine the influence of socio‐demographic factors on the level of knowledge, attitude, and practice regarding *J. heterocarpa* utilization. The findings indicated inadequate knowledge (mean score 48.6 ± 12.1) and moderately favorable attitudes (51.4 ± 6.5) and practices (61.4 ± 6.7) concerning the utilization of *J. heterocarpa.* Knowledge exhibited a positive correlation with practices (*r* = 0.535, *p* < 0.01). Knowledge and practices demonstrated a negative correlation with the awareness of health benefits (*r* = −0.625, *p* < 0.01 and *r* = −0.464, *p* < 0.01, respectively). The age of individuals significantly affected respondent's understanding of therapeutic properties and the decline of *J. heterocarpa* in its usual frequented growing areas (*p* < 0.05), emphasizing the crucial role of older generations in preserving traditional knowledge. Utilization of *J. heterocarpa* is significantly constrained by cultural beliefs such as seasonality (93.1%) as well as its association with snakebite treatment (57%) which hinders victims from ever consuming the vegetable. The limited knowledge of *J. heterocarpa*, combined with moderate attitudes and practices, restricts its potential contribution to food security. Consequently, targeted interventions that promote knowledge, sustainable practices, and culturally sensitive approaches are essential for optimizing the benefits of the plant utilization.

## Graphical abstract

This study assessed Knowledge, Attitude and Practices (KAP) of the Waluguru community towards *Justicia heterocarpa*. Findings revealed a moderately positive attitude and practices but limited utilization due to a significant knowledge gap in cultivation and post‐harvest handling, as well as socio‐cultural barriers. The study recommends culturally sensitive and awareness interventions that will promote sustainable practices to enhance the vegetable's contribution to food security and nutrition.

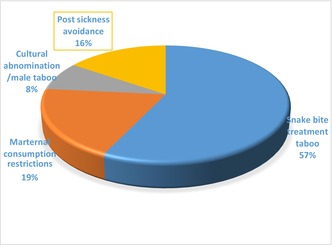

## 1 Introduction

Micronutrient deficiencies often referred to as hidden hunger persist as a critical public health burden in low and middle‐income countries (LMICs), affecting more than 2 billion people worldwide despite adequate energy intakes (Li et al. 2020; Lowe 2021). Hidden hunger stems not just from economic constraints but from monotonous, nutrient‐poor diets dominated by staples lacking in essential vitamins and minerals (Jeje et al. 2023; Lowe 2021). These deficiencies especially iron, vitamin A, zinc and iodine undermine child growth, cognitive development, immune function which impose long‐term health consequences (Issa‐Zacharia et al. 2024; Kancherla et al. 2021; Li et al. 2020; Redón 2021; Stoll et al. 2021). In Tanzania, studies reported that 58% of children between the ages of 6 and 59 months have anemia (Bukenya et al. 2023), and 34% have vitamin A insufficiency (Kilasy et al. 2024; Salha et al. 2023). While alternative solutions like bio‐fortification and supplementation have been explored, dietary diversification remains one of the most sustainable approaches to combat these micronutrient deficits (Li et al. 2020; Ohanenye et al. 2021).

Indigenous Leafy Vegetables (ILVs) offer a promising avenue for dietary diversification. They are rich in essential minerals and vitamins, including Calcium (Ca), Zinc (Zn), Iron (Fe), Manganese (Mn), and pro‐vitamin A, which can help prevent chronic diseases such as diabetes, cancer, and high blood pressure (Atuna et al. 2022; Issa‐Zacharia et al. 2024; Stoll et al. 2021). ILVs are culturally significant, easily accessible, and thrive in local environments (Bokelmann et al. 2022; Borelli et al. 2020). ILVs have significantly improved food and nutrition security in sub‐Saharan Africa and several Asian countries (Imathiu 2021). Recent empirical studies from East Africa demonstrate that ILVs are integrated into daily mealsand play a vital role in enhancing dietary diversity, improving nutritional adequacy and strengthening food and nutrition security at the household level (Mwadzingeni et al. 2021; Sakamoto et al. 2022; Bokelmann et al. 2022).

This study focuses on *Justicia heterocarpa*, locally known as “mwidu.” It is a staple in the traditional diet of the Waluguru and Wapogoro communities in Morogoro, Tanzania, with the Waluguru being the primary consumers (Sangija, Martin, and Matemu 2022; Swai et al. 2025). This plant is a valuable source of several important micronutrients such as pro vitamin A (betacarotene and lutein), and minerals (iron and zinc) making it an excellent option to combat local dietary deficits (Gowele et al. 2019). According to the study conducted by Swai et al. (2025), *J. heterocarpa* is mostly collected in the wild and consumed incorporated with other indigenous vegetables like *Corchorus olitorius* (*mgunda*), okra and different types of legumes to make it the perfect dish alongside stiff porridge.

Despite the significant potential of ILVs, including *J. heterocarpa*, there is a significant limitation of information regarding their value chains, consumption patterns, and their potential uses in the literature (Imathiu, 2021; Shayanowako et al. 2021). This gap is particularly evident in rural Tanzania, where there are limited studies that evaluated the Knowledge, Attitudes, and Practices regarding the ILVs value chain (Moyo et al. 2022).

This study aims to assess community perceptions and identify usability challenges of *J. heterocarpa* by evaluating the knowledge, attitude, and practices (KAP) of the Waluguru community in Kiroka Ward. Kiroka Ward was selected due to the plant's high abundance, its availability in local markets, and the community's significant cultural connections to its use, as it functions as a central hub for the plant's consumption (Sangija, Martin, and Matemu 2022). Despite its cultural significance, a gap persists in the scientific understanding of the plant, contributing to challenges within its value chain. This study seeks to address this gap by offering a thorough evaluation of the community's knowledge, attitudes, and practices (KAP) along with the related obstacles to complete utilization. The collected data will inform evidence‐based recommendations aimed at improving consumption, bolstering food security, and advancing dietary diversity and sustainable agriculture.

## 2 Material and Methods

**FIGURE 1:**
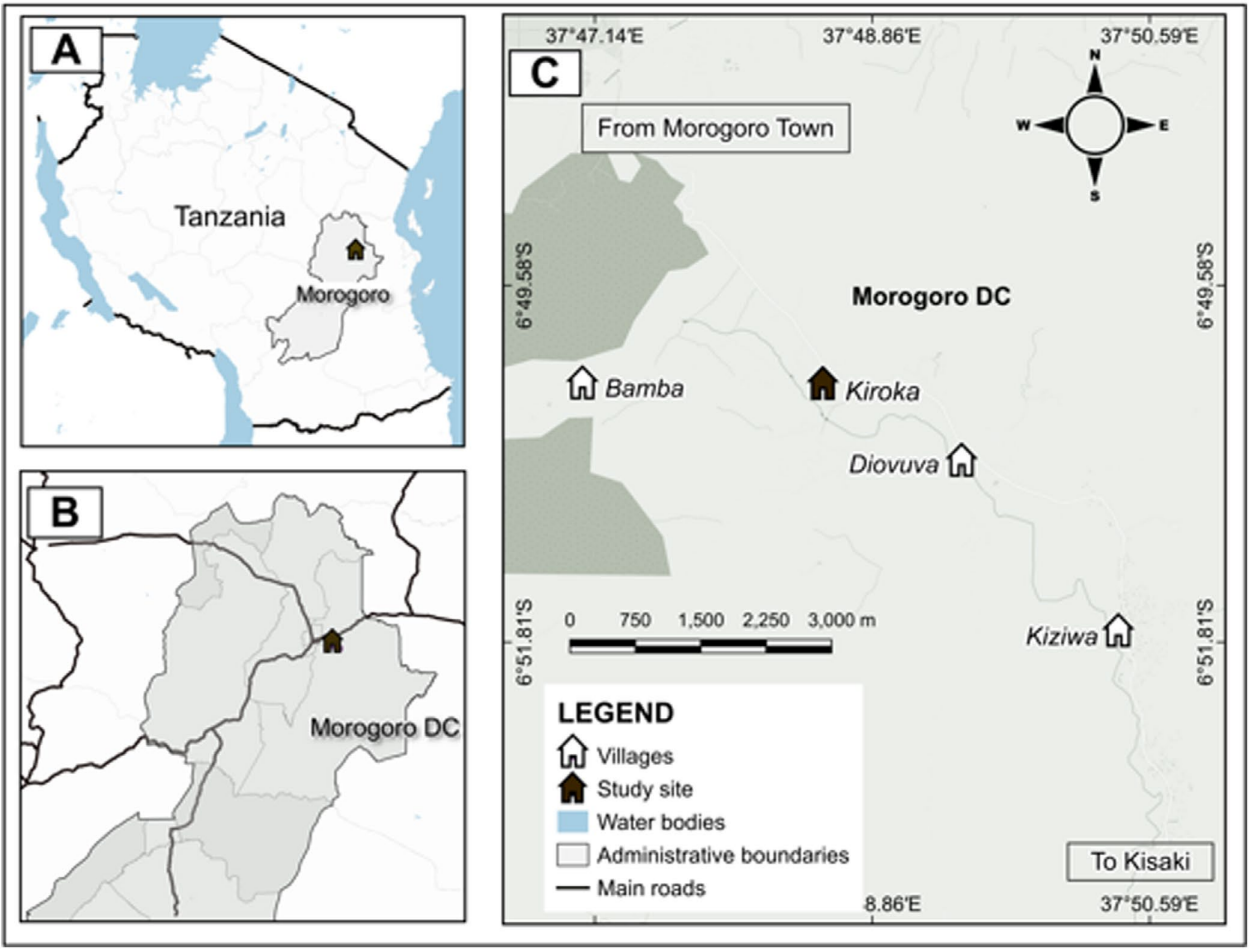
Map of the study area showing the location of Kiroka Ward and its four constituent villages (Kiroka, Kiziwa, Diovuva, and Bamba) within Morogoro Rural, Tanzania.

Morogoro Rural constitutes one of the six districts within the Morogoro Region of Tanzania. The district consists of six wards. Kiroka ward consists of four villages which are Kiroka, Kiziwa, Diovuva and Bamba as shown in Figure 1. The choice of Kiroka village as the research site was intentional and strategically fit with the study's aims. Kiroka Village hosted a substantial population of the Waluguru tribe, the ethnic group of principal focus for this study. The Waluguru people traditionally regarded *J. heterocarpa* as an indigenous vegetable, and its eating was integral to their cultural practices. Kiroka village served as an optimal site to examine the particular interconnections among the Waluguru tribe, their agricultural techniques, and the use of this culturally significant vegetable.

### 2.1 Study Design

A cross‐sectional design was employed to assess the knowledge, attitude and practices towards the utilization of *J. heterocarpa* among the households in Kiroka village.

### 2.2 Study Population

The method of sampling utilized was snowball sampling. The Village Extension Officer (VEO) initially facilitated the identification of households that satisfied the inclusion criteria for participation, specifically those aged 18 years and older who are familiar with the consumption and growth of *J. heterocarpa*. After the initial purposive identification of “seed” participants, subsequent respondents were recruited via referrals, with participating household members identifying neighboring households that also consumed the vegetable. The chain‐referral process persisted until the target sample size was achieved. It is important to note that certain community members opted not to participate in the survey due to cultural taboos that discourage individuals who have utilized the plant for medicinal purposes, such as for snakebite or malaria, as well as pregnant women, from consuming it as a food source.

### 2.3 Sample Size and Sampling Procedures

This exploratory study employed purposive sampling to select respondents with experience in *J. heterocarpa* utilization. Cochran's formula was used to calculate the ideal sample size based on a 95% confidence level and a 5% margin of error. The appropriate sample size.

$$\mathrm{no}=\frac{z^{2}\times p\times \left(1-p\right)}{e^{2}}=\frac{{\left(1.96\right)}^{2}\times 0.5\times \left(1-0.5\right)}{0.05^{2}}$$

The calculation yielded a target sample size of 384 participants. However due to practical and logistical constraints, including limited funding and inherent challenges of accessing a culturally defined population, the final sample was adjusted using the formula for a finite population $n=\frac{n_{0}}{1+\left(\frac{n_{0-1}}{N}\right)}$ The final sample size for the study was 174 participants. *n* = Adjusted sample size for the finite population. *N* = total population size.

This was necessary as some community members were unwilling to participate due to cultural restrictions.

### 2.4 Data Collection Tools and Methods

Data was collected by using a structured questionnaire. Verbal and written consent was obtained prior to participation in the study. The questions were developed based on relevant literature (Kirui et al. 2023; Kavoi and Kimambo 2021) with some modifications.

A pilot study was conducted to pre‐test the questionnaire with 20 participants from the waluguru community residing near Sokoine University of Agriculture. The location was chosen as it provided convenient access to members of the same target population, ensuring the questionnaire's clarity, comprehensibility, and cultural relevance which helped to refine the questions. The final questionnaire comprised five sections: demographic characteristics (age, gender) knowledge, attitude, practices, and challenges towards the utilization of *J. heterocarpa*. In addition to assessing knowledge, attitudes, and practices related to *J. heterocarpa*, the survey instrument also included questions to identify and rank other commonly consumed indigenous leafy vegetables (ILVs) in the study area. This secondary data collection was crucial for contextualizing the role of *J. heterocarpa* within the broader local food system and understanding the competitive landscape of ILV consumption preferences. Within the practice section, a single open‐ended question was included to allow respondents to state any known cultural restrictions or food taboos related to *J. heterocarpa* consumption. The qualitative responses were then categorized into key themes through a thematic analysis to determine the prevalence of each restriction. Data collection took place over 4 weeks, with interviews conducted at respondents' households.

#### 2.4.1 Variable and Measurements

##### 2.4.1.1 Knowledge

Knowledge was assessed through multiple‐choice questions, with correct answers awarded 1 point and incorrect or unclear responses scoring 0 points Include questions that gauged their awareness regarding the cultivation, post‐harvest handling, processing and benefits of the vegetable. Use closed‐ended multiple‐choice questions to capture knowledge. Example: what are the health benefits of consuming *J. heterocarpa*? (Check all that apply).

##### 2.4.1.2 Attitude

The attitude dimension included statements evaluated on a five‐point Likert scale, ranging from strongly agree (5 points) to strongly disagree (1 point) as reported by Kavoi and Kimambo (2021) with some modifications. Use Likert‐scale questions to gauge respondents' attitudes towards the vegetable (e.g., 1 = Strongly Disagree, 5 = Strongly Agree). For example, Indigenous vegetables including *J. heterocarpa* are for the low economic people, old‐fashioned and food for elderly people.

##### 2.4.1.3 Practice

The practice dimension was evaluated by using multiple choice questions. Responses indicating a positive practice were awarded 1 point, while responses indicating an absence of the practice or I don't know were scored zero points. Example: How often do you consume *J. heterocarpa* during the wet season?. Responses such as “frequently” or “regularly” were awarded 1 point, while “never” received zero points.

Constraints of utilization of *J. heterocarpa*: the questions were structured to determine the presence or absence of a specific challenge, which is a binary “Yes” or “No” response. For example, a respondent was asked, “Is there a scarcity of this vegetable during the dry season?”. The answer was recorded as either “Yes” (indicating the challenge is present) or “No” (indicating it is not).

##### 2.4.1.4 Demographics

Collect demographic data (e.g., age, gender, occupation, education) to allow for analysis of KAP patterns across different groups. Explanatory factors such as awareness of health benefits of the vegetable and training opportunities were added as independent variables along with demographic factors to investigate their influence on categorical KAP scores.

The scores for KAP were each converted to a 100‐point scale to facilitate comparison and interpretation as described by O'Donnell et al. (2022) with some modifications. The conversion was performed by dividing the total number of points obtained by respondent by the maximum possible score for that section and then multiplying by 100 (a percentage‐based scale). A score of < 50 was considered to indicate a low, scores between 50 and 75 were considered a median (moderate) and scores > 75 as good.

### 2.5 Statistical Analysis

Data analysis was conducted using SPSS version 27.0. Descriptive statistics were used to summarize the socio‐demographic characteristics and key explanatory factors (i.e., awareness of health benefits and training) of the respondents and the distribution of KAP scores. Chi‐squared tests were employed to examine the associations between categorical socio‐demographic factors and explanatory factors and categorical KAP score levels. Furthermore, the chi‐square test was used (binary categorical YES/NO) to test constraints of utilization of *J. heterocarpa* and their frequency of occurrence. Pearson correlation coefficients were calculated to assess the linear relationships between continuous KAP scores and continuous socio‐demographic variables. Ordinal regression models were used to examine the influence of socio‐demographic factors and key explanatory factors on the level of knowledge, attitude, and practice regarding *J. heterocarpa* utilization.

### 2.6 Ethical Considerations

Ethical approval for conducting the current study was obtained from Sokoine University of Agriculture and from the government Permanent Secretary through Regional Administration and Local Government in Dodoma. Additionally, authorization to carry out surveys in Kiroka Ward was obtained from the District and Regional Government Authorities. Only participants aged 18 years and above were recruited into the study as confirmed by a verbal screening question at the beginning of the interview. Participants were informed about the study purpose, and confidentiality was maintained throughout. Verbal informed consent was secured from every participant prior to data collectionand parental consent for minors was not applicable as no individuals under the age of 18 years were included. Participation was voluntary and respondents were informed that they could withdraw at any time during the study.

## 3 Results

### 3.1 Socio‐Demographic Characteristics of Respondents in Kiroka Morogoro Rural

Table 1 presents the socio‐demographic characteristics of the respondents. Over half (54.6%) of the participants were married, and a significant majority (67%) were female. A notable proportion of respondents (77.5%) fell within the age range of 29–39 years while most households (79.3%) comprised one to five individuals. Regarding education, 60.9% of participants had completed primary school education. Furthermore, the predominant occupation among respondents (92%) was in agriculture or small business.

**TABLE 1:** Demographic and socio‐economic characteristics of respondents.

Variable	Categorical	Frequency	Percent (%)
Sex of respondent	Female	107	61.50
	Male	67	38.50
Name of division	Temekelo A	72	41.40
	Temekelo B	65	37.40
	Temekelo C	37	21.30
House hold size	1–5	138	79.30
	6–10	36	20.70
Age of respondent	18–28	34	19.50
	29–39	66	37.90
	40–50	35	20.10
	51–61	16	9.20
	62+	23	13.20
Education level	I do not attend school	28	16.10
	Primary education	106	60.90
	Secondary education	35	20.10
	Diploma	3	1.70
	Degree	2	1.10
Marital status	Married	95	54.60
	Widowed/widower	16	9.20
	Divorced	11	6.30
	Separated	6	3.40
	Single	46	26.40
Occupation	None	11	6.30
	Farmer	75	43.10
	I own small business	85	48.90
	Government employee	2	1.10
	Non‐government employee	1	0.60
Are you the head of household	Yes	101	58.00
	No	73	42.00

*Note:* Socio‐demographic data are presented as frequency (F) and percentage (%). The total number of participants included in the analysis is *n* = 174.

### 3.2 The Respondents' Knowledge Regarding the Cultivation, Post‐Harvest Handling, and Utilization of *J. heterocarpa*

Table 2 presents the respondents' knowledge on various aspects of *J. heterocarpa* cultivation, post‐harvest handling and utilization. Chi‐squared tests were conducted to assess the association between the age category of the respondents and their responses to each knowledge question. A significant association was revealed between perceived health benefits for gastrointestinal issues (*p* = 0.025) and the age category of respondents. This suggests that different age groups hold significantly different views on knowing the health benefits. However, there was a significant association between the age category and their perception of what the main causes of the decline of *J. heterocarpa* in its customarily frequented areas were. The results indicated that different age groups had different opinions on what drove the decline Only 47.3% of the respondents correctly identified caterpillars, whiteflies, and aphids as frequent pests. Most respondents (98.4%) were unaware of various processing techniques such as fermentation, blanching, and solar drying. Very few respondents (6%) directly engaged in sun drying *J. heterocarpa* for future use. This process involved placing the vegetable directly in the sun, which has been shown to degrade the nutritional composition (nutrients) that are naturally present in the vegetable. Approximately 81% of respondents lacked awareness regarding optimal packaging materials that enhance the shelf life of vegetables. The majority of the respondents (89.1%) lacked awareness regarding the nutrients present in *J. heterocarpa*. Similarly, 77% of respondents lacked awareness of its health benefits. Most of the ILVs including *J. heterocarpa* hold a therapeutic value because these vegetables contained a high amount of antioxidants with medicinal properties. About 35.6% of respondents believed that *J. heterocarpa* could cure diseases. Specific medicinal applications, such as treating gastrointestinal problems (28.7%) and anemia (29.3%), were acknowledged by a smaller proportion of participants, whereas a notable 66.7% believed vegetables could treat snake bites by applying it externally as a paste Most participants (74.1%) reported a significant decrease in the availability of *J. heterocarpa* in commonly visited locations. The Pearson chi‐square analysis indicated a significant association between age and knowledge of the therapeutic properties of vegetables, such as their effectiveness in curing snake bites (*p* = 0.043) and gastrointestinal issues (*p* = 0.025). Age demonstrated a significant relationship with awareness regarding the decline of *J. heterocarpa* in its typical habitats, attributed to urbanization and climate change (*p* = 0.02). The findings underscore the influence of age on the development of awareness regarding health and environmental issues.

**TABLE 2:** Respondents' knowledge on vegetable cultivation, post‐harvest handling and utilization.

Variable	Categories	Frequency	Percent	Chi‐squared	*p*
Which of the following is a common pest that affects this vegetable?	Caterpillars	30	17.20	9.366	0.671
	Aphids	13	7.50		
	Caterpillars, white flies, aphids	39	22.40		
	I do not know	92	52.90		
What do you do immediately after harvesting the vegetable?	Spread under shade	44	25.30	10.943	0.205
	Pack in containers	96	55.20		
	Wash with clean water	0	0.00		
	Warp in banana leaves	34	19.50		
What is the best method to process this vegetable to increase its shelf life?	Sun‐drying the leaves	9	5.20	3.724	0.445
	Blanching the vegetable	0	0.00		
	Fermenting the vegetable	0	0.00		
	I do not know	165	94.80		
Have you received any training on post‐harvest handling ILVs?	Yes	0	0.00		
	No	174	100.00		
	I do not know	0	0.00		
Which of the following nutrients is the mostly available in *J. heterocarpa*?	Vitamin A	0	0.00	7.642	0.469
	Iron	8	4.60		
	Calcium	11	6.30		
	I do not know	155	89.10		
Does consuming *J. heterocarpa* has health benefits?	No	0	0.00	2.5	0.645
	Yes	40	23.00		
	I do not know	134	77.00		
Do you believe this vegetable has medicinal properties?	Yes	62	35.60	9.53	0.3
	No	68	39.10		
	I do not know	44	25.30		
Gastrointestinal	No	76	43.70	17.515	0.025
	Yes	50	28.70		
	I do not know	48	27.60		
Anemia (low iron)	No	123	70.70	3.085	0.544
	Yes	51	29.30		
Cancer	No	113	64.90	5.585	0.694
	Yes	20	11.50		
	I do not know	41	23.60		
Snake bite	No	17	9.80	15.944	0.043
	Yes	116	66.70		
	I do not know	41	23.60		
Decline of the plant in its customarily frequented area	No	45	25.90	8.662	0.07
	Yes	129	74.10		
Main causes of its decline	Urban expansion	20	11.60	24.125	0.02
	Deforestation	42	24.40		
	Climate change	65	37.80		
	I do not know	45	26.20		

*Note:* All results are based on *n* = 174 participants. Data are presented as frequency and percentage (%). The Chi‐squared analysis tests the association between the age category and the responses to each knowledge question.

Table 3 presents the association between socio‐demographic characteristics and the categorized knowledge scores (low, medium, good) of the respondents as well as the effect of these characteristics on the likelihood of being in a higher knowledge category by using ordinal logistic regression. In the ordinal regression (*p* = 0.016), the results on household sizes (*p* = 0.016) revealed statistical significance suggested that households with 6 to 10 people had a higher median knowledge score than those with less members. Furthermore, age served as a predictor of knowledge scores (*p* = 0.031), with younger respondents attaining a medium knowledge score of 61.8%, which exceeded that of older respondents. Individuals with higher education qualifications, including diplomas or degrees, exhibited inadequate knowledge scores. The recognition of health benefits significantly influenced knowledge (*p* < 0.001), indicating a correlation between understanding of the vegetable's advantages and the level of knowledge acquired. However, the table revealed having received training on the value chain of indigenous vegetables had a statistically significant effect (*p* < 0.05 on the odds of a respondent being in a higher knowledge category).

**TABLE 3:** Effect and association of socio‐demographic characteristics and explanatory factors on the knowledge scores categories.

Characteristics	Knowledge score (number of respondents [%])			*p* ^+^	Mean ± SD	Range	*p*
	Low	Medium	Good				
House hold size							
1–5	76 (55.1%)	61 (44.2%)	1 (0.7%)	0.071	48.4 ± 12.23	25–82.14	0.016
6–10	15 (41.7%)	19 (52.8%)	2 (5.6%)		49.5 ± 11.74	21.43–71.43	
11–15	0 (0%)	0 (0%)	0 (0%)				
Age of respondent (years)							
18–28	12 (35.3%)	21 (61.8%)	1 (2.9%)	0.366	50.84 ± 12.65	28.57–82.14	0.031
29–39	38 (57.6%)	27 (40.9%)	1 (1.5%)		46.97 ± 11.32	28.57–75	
40–50	20 (57.1%)	15 (42.9%)	0 (0%)		52.55 ± 11.08	32.14–71.43	
51–61	7 (43.8%)	8 (50%)	1 (6.3%)		45.98 ± 13.16	25–71.43	
62+	14 (60.9%)	9 (39.1%)	0 (0%)		45.96 ± 13.16	21.43–71.43	
Education level							
Not attend school	12 (42.9%)	15 (53.6%)	1 (3.6%)	0.826	52.3 ± 12.03	25–71.43	0.603
Primary education	55 (51.9%)	49 (46.2%)	2 (1.9%)		47.71 ± 11.59	21.43–75	
Secondary education	20 (57.1%)	15 (42.9%)	0 (0%)		48.06 ± 13.98	28.57–82.14	
Diploma	2 (66.7%)	1 (33.3%)	0 (0%)		52.38 ± 8.25	42.86–57.14	
Degree	2 (100%)	0 (0%)	0 (0%)		50 ± 5.05	46.43–53.57	
Marital status							
Married	52 (54.7%)	42 (44.2%)	1 (1.1%)	0.681	48.83 ± 11.96	28.57–75	0.03
Widowed/widower	10 (62.5%)	6 (37.5%)	0 (0%)		49.78 ± 13.89	25–71.43	
Divorced	4 (36.4%)	7 (63.6%)	0 (0%)		50.65 ± 12.56	32.14–67.86	
Separated	2 (33.3%)	4 (66.7%)	0 (0%)		47.62 ± 7.72	39.29–60.71	
Single	23 (50%)	21 (45.7%)	2 (4.3%)		47.44 ± 12.46	21.43–82.14	
Training							
Yes	0 (0%)	7 (100%)	0 (0%)	0.014	50 ± 16.88	21.43–71.43	0.022
No	91 (54.5%)	73 (43.7%)	3 (1.8%)		48.57 ± 11.93	25–82.14	
Health benefit							
No	0 (0%)	0 (0%)	0 (0%)	< 0.001			< 0.001
Yes	10 (23.8%)	29 (69%)	3 (7.1%)		61.99 ± 9.46	42.86–82.14	
I do not know	81 (61.z4%)	51 (38.6%)	0 (0%)		44.37 ± 9.49	21.43–67.86	

*Note:* All results are based on *n* = 174 respondents. Data are presented as Mean ± SD (used for error bars) and range. The *p* ^+^‐value tests the categorical association using Chi‐squared analysis, while the final *p*‐value is derived from a regression analysis determining the predictive relationship.

### 3.3 Attitude of Respondents Towards Value Chain of *J. heterocarrpa*

Table 4 presents respondents' attitudes towards the *J. heterocarpa* value chain. For each statement (A‐K), the frequency and percentage of responses across a five‐point Likert scale were displayed. However, the association between the attitude statements and the status of being a head of household was also assessed. There was a statistically significant association between household head status and the statement, “cultivation and processing of *J. heterocarpa* are primarily activities undertaken by women” (*p* = 0.027, mean = 4.13). This result highlights the gendered nature of this indigenous vegetable, indicating that women served as the leading guardians of the value chain. Conversely, a significant portion of respondents disagreed (60.9%) or strongly disagreed about the high demand for value‐added products of *J. heterocarpa*, such as dried or fermented forms (*p* = 0.003, mean = 2.28). The impression of low demand for processed goods points to a lack of consumer interest in the processed ILVs. For all attitude statements (A, B, C, F, G, H, I and K) there was no statistical significance (*p* > 0.05). Eighty‐five per cent of the respondents (mean = 4.19), agreed or strongly agreed that local farmers would benefit from growing *J. heterocarpa*. The knowledge of the vegetable's contribution to increasing food security suggests a possible influence on its acceptance for production. Regarding the presence of enough market demand for *J. heterocarpa* (*p* = 0.058, mean = 3.14), the replies were either agreed (17.2%) or neutral (47.7%). This suggests ambiguity about market possibilities. There is a need to strengthen market linkages and promote vegetables. Furthermore, a significant proportion of respondents (75.3%) disagreed or strongly disagreed that *J. heterocarpa* is solely a vegetable for impoverished individuals, associated with traditional lifestyles and older generations. This implies that the vegetable offers chances for more significant popularity since it has excellent versatility and nutritional value fit for the people. On the other hand, most of the respondents (85.1%) opposed or strongly disagreed with the claim that this vegetable's taste, look, and quality are less than those of exotic vegetables, therefore suggesting a reasonable opinion of the vegetable.

**TABLE 4:** Attitudes towards value chain of *J. heterocarpa*.

Variable	Strongly disagree	Disagree	Neutral	Agree	Strongly agree	Chi squared	*p*	Mean
A	3 (1.7%)	10 (5.7%)	21 (12.1%)	57 (32.8%)	83 (47.7%)	5.417	0.247	4.19
B	93 (53.4%)	33 (19%)	18 (10.3%)	20 (11.5%)	10 (5.7%)	2.116	0.715	1.97
C	0 (0%)	14 (8%)	82 (47.1%)	65 (37.4%)	13 (7.5%)	2.544	0.467	3.44
D	0 (0%)	12 (6.9%)	23 (13.2%)	70 (40.2%)	69 (39.7%)	9.142	0.027	4.13
E	44 (25.3%)	62 (35.6%)	44 (25.3%)	24 (13.8%)	0 (0%)	13.822	0.003	2.28
F	91 (52.3%)	40 (23%)	43 (24.7%)	0 (0%)	0 (0%)	3.875	0.144	1.72
G	118 (67.8%)	56 (32.2%)	0 (0%)	0 (0%)	0 (0%)	2.184	0.139	1.32
H	61 (35.1%)	87 (50%)	10 (5.7%)	16 (9.2%)	0 (0%)	1.164	0.762	1.89
I	52 (29.9%)	69 (39.7%)	17 (9.8%)	27 (15.5%)	9 (5.2%)	0.988	0.912	2.26
J	32 (18.4%)	0 (0%)	83 (47.7%)	30 (17.2%)	29 (16.7%)	7.466	0.058	3.14
K	72 (41.4%)	70 (40.2%)	10 (5.7%)	20 (11.5%)	2 (1.1%)	5.8	0.215	1.908

*Note:* All results are based on *n* = 174 respondents. Data are presented as Frequency (F) and percentage (%) across the five‐point Likert scale. The Chi‐squared value and *p*‐value test the statistical association between Household Head Status and the responses to each attitude statement. A, cultivating indigenous vegetable is beneficial for local farmers; B, cultivating *J. heterocarpa* requires a lot of resources and effort; C, proper storage techniques of raw *J. heterocarpa* can significantly extend the shelf life of this vegetable; D, *J. heterocarpa* cultivation, collection, processing and cooking is a woman's activity; E, value‐added products (e.g., dried or processed forms) of this vegetable are in high demand; F, *J. heterocarpa* is the vegetable for low economic people, traditional lifestyle and food for older generation; G, this vegetable is versatile and can be used in a variety of dishes; H, the taste, appearance and quality of this vegetable are not as good as that of exotic vegetables; I, too much eating of *J. heterocarpa* causes health problems; J, there is enough market demand for this vegetable; K, proper packaging of this vegetable can increase its price/sells.

Table 5 presents the association between socio‐demographic characteristics and the categorized attitude scores (low, medium good) of the respondents as well as the effect of these characteristics on the likelihood of being in a higher attitude category by using ordinal logistic regression. The education level demonstrates a statistically significant association with attitude level (Chi‐squared, *p* = 0.023); nevertheless, the limited number of respondents with higher education complicates the ability to draw definitive conclusions regarding the influence of education on positive attitude. A more positive attitude is linked to awareness of the health benefits of *J. heterocarpa* (Chi‐squared, *p* = 0.03; Regression, *p* = 0.009). With women showing a more positive attitude than men (*p* = 0.049), the sex of a person has a minor impact on the attitude towards vegetable use.

**TABLE 5:** Effect of socio‐demographic characteristics on attitude levels and their determinants among respondents.

Characteristics	Attitude score (number of respondents [%])			*p* ^+^	Mean ± SD	Range	*p*
	Low	Medium	Good				
Sex of respondent							
Female	47 (43.9%)	60 (56.1%)	0 (0%)	0.493	52 ± 6.65	36.36–69.09	0.049
Male	33 (49.3%)	34 (50.7%)	0 (0%)		50.37 ± 6.22	36.36–63.64	
Age of respondent							
18–28	15 (44.1%)	19 (55.9%)	0 (0%)	0.539	52.14 ± 6.45	40–63.64	0.069
29–39	33 (50%)	33 (50%)	0 (0%)		51.35 ± 6.11	40–65.45	
40–50	12 (34.3%)	23 (65.7%)	0 (0%)		52.88 ± 7.59	36.36–69.09	
51–61	9 (56.3%)	7 (43.8%)	0 (0%)		48.98 ± 5.88	36.36–56.36	
62+	11 (47.8%)	12 (52.2%)	0 (0%)		49.64 ± 6.09	38.18–63.64	
Education level							
Did not attend school	7 (25%)	21 (75%)	0 (0%)	0.024	53.77 ± 6.86	36.36–69.09	0.076
Primary education	54 (50.9%)	52 (49.1%)	0 (0%)		50.99 ± 6.67	36.36–65.45	
Secondary education	19 (54.3%)	16 (45.7%)	0 (0%)		50.18 ± 5.79	38.18–61.82	
Diploma	0 (0%)	3 (100%)	0 (0%)		54.55	54.55–54.55	
Degree	0 (0%)	2 (100%)	0 (0%)		53.64 ± 1.29	52.73–54.55	
Marital status							
Married	42 (44.2%)	53 (55.8%)	0 (0%)	0.691	51.6 ± 5.93	38.18–65.45	0.491
Widowed/widower	7 (43.8%)	9 (56.3%)	0 (0%)		50.34 ± 6.15	36.36–58.18	
Divorced	4 (36.4%)	7 (63.6%)	0 (0%)		52.73 ± 7.54	36.36–63.64	
Separated	2 (33.3%)	4 (66.7%)	0 (0%)		53.94 ± 8.27	41.82–61.82	
Single	25 (54.3%)	21 (45.7%)	0 (0%)		50.59 ± 7.41	40–69.09	
Occupation							
None	7 (63.6%)	4 (36.4%)	0 (0%)	0.651	48.76 ± 3.88	40–54.55	0.397
Farmer	35 (46.7%)	40 (53.3%)	0 (0%)		51.49 ± 7.2	36.36–69.09	
Owns small business	37 (43.5%)	48 (56.5%)	0 (0%)		51.66 ± 6.22	38.18–65.45	
Government employee	1 (50%)	1 (50%)	0 (0%)		48.18 ± 3.86	45.45–50.91	
Non‐government employee	0 (0%)	1 (100%)	0 (0%)		52.73	52.73–52.73	
Known health benefit							
No	0 (0%)	0 (0%)	0 (0%)	0.003			0.009
Yes	11 (26.2%)	31 (73.8%)	0 (0%)		53.38 ± 4.3	47.27–61.82	
I do not know	69 (52.3%)	63 (47.7%)	0 (0%)		50.73 ± 6.97	36.36–69.09	

*Note:* All results are based on *n* = 174 respondents. Data are presented as Mean ± SD (used for error bars) and range. The *p* ^+^‐value tests the categorical association using Chi‐squared analysis, while the final *p*‐value is derived from a regression analysis determining the predictive relationship.

### 3.4 Respondents' Practices Towards Utilization of *J. heterocarpa*

Table 6 illustrates the respondents' practices regarding the value chain of *J. heterocarpa*. A significant 68.4% of respondents do not collect or store *J. heterocarpa* seeds for domestication, while only 8.6% consistently broadcast the seeds in their gardens. The limited domestication of the plant can be attributed to its primary growth in wild environments. The vegetable exhibits significant perishability, with 65.5% indicating that optimal storage conditions allow for a period of approximately 1–2 days. This situation highlighted the necessity for enhanced post‐harvest management methods, including value addition. 93.1% of the interviewees do not engage in vegetable processing. While 6.9% of respondents utilize direct sun drying, only 10.3% consume *J. heterocarpa* in the off‐season. During periods of surplus, 51.8% consume it either daily or up to 2 to 3 days per week. A majority of respondents (85.6%) indicated that the vegetable is preferred by all family members. 80.5% of respondents indicated that *J. heterocarpa* is utilized in traditional ceremonies, such as weddings and puberty education for girls, highlighting the vegetable's significance within the community.

**TABLE 6:** Practices on cultivation, handling, consumption and utilization of *J. heterocarpa*.

Variable	Categories	Frequency	Percent (%)
Seeds for home garden	Never	119	68.40
	Occasionally	40	23.00
	Regularly	15	8.60
Pest control use type	Organic methods (ash, etc.)	0	0.00
	Chemical pesticides	0	0.00
	No pest control	55	31.60
	It grows wild	119	68.40
How long can the vegetable be stored after harvesting before it spoils	Less than a day	0	0.00
	1–2 days	114	65.50
	3–5 days if properly stored	24	13.80
	I do not know	36	20.70
Which of the following processing methods do you use for the *J. heterocarpa*?	Direct sun Drying	12	6.90
	Solar drying	0	0.00
	Fermentation	0	0.00
	I do not do any processing	162	93.10
How do you usually cook the vegetable	Boil with okra and legumes	118	67.80
	Boil with okra alone	42	24.10
	Stir‐fried with oil and spices	14	8.00
How many minutes do you cook your *J. heterocarpa*?	1–5 min	70	40.20
	6–10 min	58	33.30
	11–20 min	42	24.10
	Half an hour	4	2.30
Which foods are eaten alongside with this vegetable?	Stiff porridge (ugali)	158	90.80
	Rice	12	6.90
	I eat it alone	4	2.30
Selling fresh vegetable	Local village markets	21	12.10
	Regional town markets	23	13.20
	Directly to consumers	16	9.20
	Not sure, I do not sell	114	65.50
How often do you consume *J. heterocarpa*	Daily	17	9.80
	2–3 times per week	73	42.00
	4–6 times per week	51	29.30
	Once a week	23	13.20
	Rarely	10	5.70
Consumption in dry season	Yes	44	25.30
	No	130	74.70
Preference in HH	The whole family	149	85.60
	Children under 18 years	24	13.80
	Me during pregnancy	1	0.60
	My husband	0	0.00
What part of the vegetable do you usually consume	Leaves only	48	27.60
	Leaves stems and flower	116	66.70
	Whole plant	10	5.70
	I do not know	0	0.00
	Yes	140	80.50
Types of traditional ceremonies	Wedding ceremonies	23	13.20
	Educative ceremonies, puberty girls	117	67.20
	I do not know	34	19.50
Restrictions hindering consumption	No	33	19.00
	Yes	116	66.70
	I do not know	25	14.40
When *J. heterocarpa* is consumed mostly?	During the rainy season	156	89.70
	During a dry season	0	0.00
	Throughout the year	18	10.30
	I do not know	0	0.00

*Note:* All practices data are presented as frequency (F) and percentage (%) based on responses from *n* = 174 participants.

Table 7 presents a relationship between socio‐demographic characteristics and respondents' practice scores categorized as (low, medium and good); furthermore it displayed the ordinal logistic regression examining the likelihood of respondents being in a higher practice score category. The descriptive statistics such as average practice score (mean with standard deviations), percentage scores and the range were also analyzed. Age significantly predicted practice scores, as indicated by ordinal regression analysis (*p* = 0.011). Individuals aged 18–28 years exhibited the highest proportion of good practice scores at 8.8%, with 85.3% achieving medium scores, resulting in a mean score of 61.51 ± 7.97 points. Awareness of health benefits of the native vegetable has a significant impact on the practice levels (*p* < 0.001). Gender does not serve as a predictor of practice scores, as indicated by the absence of statistical significance in ordinal regression and association (*p* = 0.308, *p* = 0.484) The findings showed no significant association between education level and practice scores (*p* = 0.363) indicating education level cannot affect the behavior towards *J. heterocarpa* consumption and utilization.

**TABLE 7:** Influence and associations of socio demographic characteristics on practice scores.

Characteristics	Practice score (number of respondents [%])			*p* ^+^	Mean ± SD	Range	*p*
	Low	Medium	Good				
Sex of respondent							
Female	5 (4.7%)	100 (93.5%)	2 (1.9%)	0.484	61.42 ± 6.7	44.68–76.6	0.308
Male	1 (1.5%)	64 (95.5%)	2 (3%)		61.54 ± 6.77	44.68–82.98	
Age of respondent							
18–28	2 (5.9%)	29 (85.3%)	3 (8.8%)	0.169	61.51 ± 7.97	44.68–82.98	0.011
29–39	1 (1.5%)	65 (98.5%)	0 (0%)		60.93 ± 5.87	48.94–74.47	
40–50	1 (2.9%)	34 (97.1%)	0 (0%)		63.16 ± 5.76	48.94–74.47	
51–61	1 (6.3%)	15 (93.8%)	0 (0%)		60.64 ± 9.03	48.94–74.47	
62+	1 (4.3%)	21 (91.3%)	1 (4.3%)		60.96 ± 6.59	44.68–76.6	
Education level							
I do not attend school	1 (3.6%)	27 (96.4%)	0 (0%)	0.363	63.6 ± 6.16	48.94–74.47	0.171
Primary education	3 (2.8%)	102 (96.2%)	1 (0.9%)		61.32 ± 6.27	44.68–76.6	
Secondary education	2 (5.7%)	30 (85.7%)	3 (8.6%)		60.67 ± 8.21	44.68–82.98	
Diploma	0 (0%)	3 (100%)	0 (0%)		58.87 ± 6.84	51.06–63.83	
Degree	0 (0%)	2 (100%)	0 (0%)		57.45 ± 6.02	53.19–61.7	
Marital status							
Married	4 (4.2%)	89 (93.7%)	2 (2.1%)	0.934	60.94 ± 6.96	44.68–76.6	0.155
Widowed/widower	1 (6.3%)	15 (93.8%)	0 (0%)		61.97 ± 6.45	48.94–74.47	
Divorced	0 (0%)	11 (100%)	0 (0%)		63.83 ± 5.3	53.19–70.21	
Separated	0 (0%)	6 (100%)	0 (0%)		62.06 ± 5.11	55.32–70.21	
Single	1 (2.2%)	43 (93.5%)	2 (4.3%)		61.75 ± 6.82	44.68–82.98	
Occupation							
None	0 (0%)	10 (90.9%)	1 (9.1%)	0.887	64.02 ± 8.64	53.19–82.98	0.101
Farmer	2 (2.7%)	72 (96%)	1 (1.3%)		60.96 ± 5.86	44.68–76.6	
I own small business	4 (4.7%)	79 (92.9%)	2 (2.4%)		61.63 ± 7.14	44.68–76.6	
Government employee	0 (0%)	2 (100%)	0 (0%)		63.83 ± 6.02	59.57–68.09	
Non‐government employee	0 (0%)	1 (100%)	0 (0%)		53.19	53.19–53.19	
Training							
Yes	0 (0%)	6 (85.7%)	1 (14.3%)	0.088	64.74 ± 7.95	55.32–76.6	0.032
No	6 (3.6%)	158 (94.6%)	3 (1.8%)		61.33 ± 6.64	44.68–82.98	
Health benefit							
No	0 (0%)	0 (0%)	0 (0%)	0.001			< 0.001
Yes	0 (0%)	38 (90.5%)	4 (9.5%)		66.97 ± 5.99	55.32–82.98	
I do not know	6 (4.5%)	126 (95.5%)	0 (0%)		59.72 ± 5.95	44.68–74.47	

*Note:* All results are based on *n* = 174 respondents. Data are presented as Mean ± SD (used for error bars) and range. The *p* ^+^‐value tests the categorical association using Chi‐squared analysis, while the final *p*‐value is derived from a regression analysis determining the predictive relationship.

### 3.5 Mean Scores of KAP of *J. heterocarpa*

Table 8 presents the descriptive statistics for knowledge, attitude, and practice scores among 174 respondents. The knowledge score is approximately 48.62 (SD = 12.10), suggesting a low level of knowledge. The average attitude score is around 51.37 (SD = 6.52), reflecting generally moderate attitudes with low variability. The practice score was the highest when compared to knowledge and attitude at 61.47 (SD = 6.71), indicating that respondents engage in desired practices driven by other factors such as tradition, observation or specific learning behavior more frequently than their knowledge suggests.

**TABLE 8:** Mean scores for KAP of *J. heterocarpa*.

	Mean	Standard deviation
Knowledge	48.6248	12.1043
Attitude	51.3689	6.52029
Practice	61.4698	6.70668

*Note:* Results are based on *n* = 174 participants. SD is used as the measure for error bars.

### 3.6 Other Consumed ILVs Preferred in the Study Area

**FIGURE 2:**
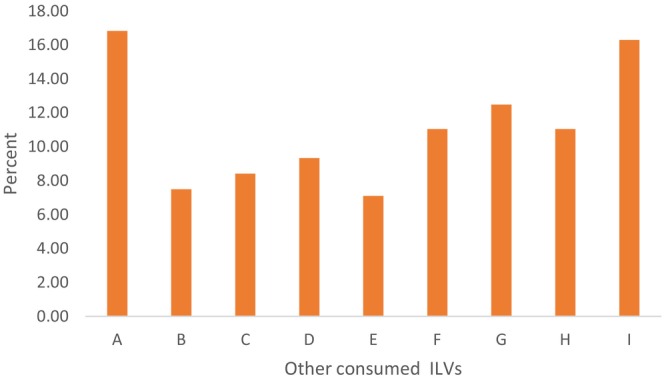
Other consumed ILVs preferred in Kiroka Ward. A, *Tembele Ipomoea* spp.; B, *Majani ya kunde* (*Vigna unguiculata*); C, Delega; D, *Mnavu* (*Solanum incanum*); E, *Mgunda* (*Corchorus olitorius*); F, *Kisamvu* (*Manihot esculenta*); G, *Majani ya maboga* (*Cucurbita pepo* L.); H, *Mchunga* (*Launea cornuta*); I, *Mchicha Amaranthus* spp.

*Ipomoea* spp. (sweet potato leaves or “Tembele”) was more preferred (16.82%) followed by *Amaranthus* spp. (“mchicha”) at 16.29% as indicated in Figure 2. A moderate consumption rate was found in *Cucurbita pepo L* (pumpkin leaves or “Majani ya maboga”) and *Manihot esculenta* (cassava leaves or “Kisamvu”). The least preference was found in *Vigna unguiculata* (cowpea leaves or “Majani ya kunde”) and *Corchorus olitorius* (“Mgunda”) High preference for sweet potato leaves and amaranth in the study area suggested that these vegetables are well established in the local diet and potentially important sources of nutrition and income for the community.

### 3.7 Cultural Restrictions and Food Taboos Related to Utilization of *J. heterocarpa*

**FIGURE 3:**
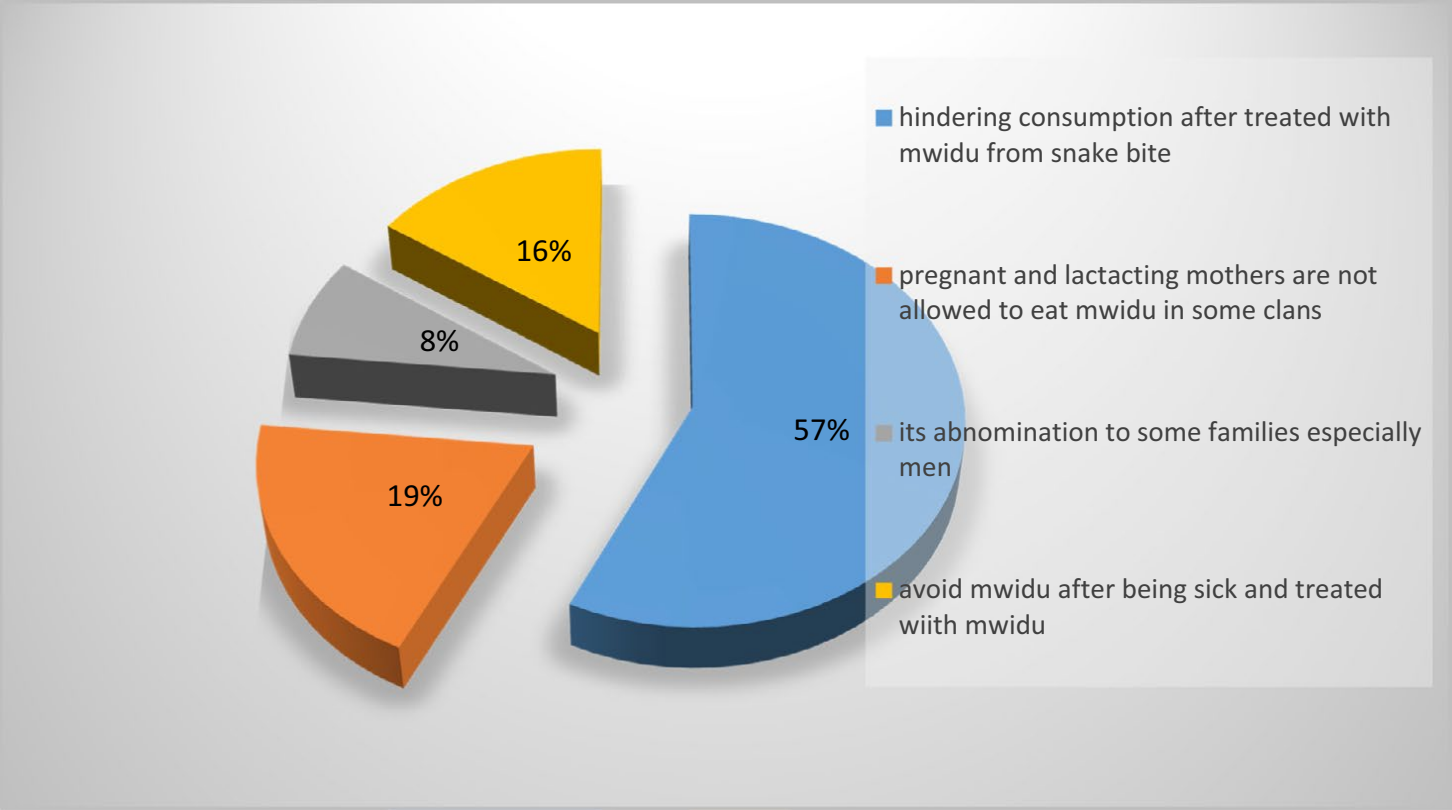
Prevalence of cultural restrictions and food taboos on *J. heterocarpa* consumption.

Figure 3 illustrates the prevalence of cultural restrictions and food taboos on the use of *J. heterocarpa*. Over half (57%) of respondents indicated that *J. heterocarpa* is limited for further consumption following the use of its leaves in snake bite treatment. Furthermore, 19% of respondents indicated that the use of *J. heterocarpa* is restricted for certain groups, including pregnant women, nursing mothers, and children under 1 year of age. Additionally, 16% of respondents indicated that individuals who have previously used *J. heterocarpa* for the treatment of conditions such as malaria or gastrointestinal disorders are prohibited from consuming it again. Individuals from certain tribes especially men (8%) are traditionally prohibited from consuming this vegetable.

### 3.8 Challenges Faced in the *J. heterocarpa* Utilization Across Its Value Chain

Table 9 presents the challenges associated with utilization of *J. heterocarpa* along its value chain for each challenge, the table showed the number and percentage of respondents who answered “No” and “Yes,” indicating whether they perceive that challenge. It also displays the Chi‐square statistic and the associated *p*‐value, which indicates the significance of the challenge's occurrence. Scarce during the dry season (*p* < 0.001) was statistically significant with the frequency of occurrence with 93.1% of respondents identified this as a significant challenge. This showed a significant seasonal dependence on plant availability, which may have restricted its consistency and market potential. The low level of domestication of the vegetable presents a significant challenge (*p* < 0.001), with 63.8% reporting this issue. The dependence on wild harvesting renders the supply susceptible to environmental influences and restricts the opportunities for regulated production and enhanced availability. The investigation and advocacy for domestication practices are essential for sustainable production. Post‐harvest losses caused by limited processing and preservation techniques during the glut season are a critical issue, with a significance level of (*p* = 0.011), impacting 77.6% of respondents. This indicated that enhanced post‐harvest management, storage and processing methods are necessary to reduce waste and optimize the economic worth of the plant. Food taboos related to the consumption of *J. heterocarpa* had a significance level of *p* < 0.001, influencing 66.1% of the participants. Additional challenges, such as eliminating conventional cultivation locations, insufficient experience or training in preservation and inadequate processing equipment, have minimal statistical significance.

**TABLE 9:** Association of challenges constrained *J. heterocarpa* utilization with their frequency of occurrence.

Variable	No	Yes	Chi‐squared	*p*
Scarce during the dry season	12 (6.9%)	162 (93.1%)	28.564	< 0.001
Lack of diversity in cooking methods	148 (85.1%)	26 (14.9%)	9.575	0.048
Unaware of Nutritional benefits	40 (23%)	134 (77%)	168.489	< 0.001
Low domestication level of the vegetable	63 (36.2%)	111 (63.8%)	130.916	< 0.001
Disappearance in its usual growth area	50 (28.7%)	124 (71.3%)	7.807	0.099
Pest and disease issues	65 (37.4%)	109 (62.6%)	19.25	0.001
High dormancy during the cold seasons	76 (43.7%)	98 (56.3%)	28.433	< 0.001
The plant easily wilt in the absence of water	54 (31%)	120 (69%)	18.041	0.001
High post‐harvest losses	39 (22.4%)	135 (77.6%)	13.007	0.011
Lack of knowledge or training in preservation	32 (18.4%)	142 (81.6%)	3.786	0.436
Insufficient equipment for processing	39 (22.4%)	135 (77.6%)	6.758	0.149
Food taboos	59 (33.9%)	115 (66.1%)	20.227	< 0.001
Termed as vegetable for poorer	141 (81%)	33 (19%)	10.195	0.037

*Note:* All results are based on *n* = 17 participants. Data are presented as binary frequencies (“No”/“Yes”). Chi‐squared analysis was used to test the statistical association between the challenge variables and the reported frequency of occurrence.

### 3.9 Correlation Coefficients Between KAP Related to *J. heterocarpa*

Table 10 displays the correlation coefficients between knowledge, attitude, and practice (KAP) scores related to *J. heterocarpa*, and various socio‐demographic characteristics. A moderate positive correlation existed between knowledge and attitude scores (*r* = 0.390. *p* < 0.01) that suggested that higher levels of knowledge are associated with more positive attitudes. However, a moderate correlation was observed between knowledge and practice scores (*r* = 0.535, *p* < 0.01) that indicated that greater knowledge is associated with better practices about the utilization of *J. heterocarpa*. A negative correlation existed between knowledge and awareness of health benefits (*r* = −0.625, *p* < 0.01). This counterintuitive finding suggests that individuals less aware of the health benefits, tend to have higher knowledge and practice scores. Perhaps those who are already knowledgeable about the plant acquired their knowledge from other sources like traditional knowledge or culinary uses which shape their behavior towards the utilization of *J. heterocarpa*. However, knowledge was positively correlated with being the head of household (*r* = 0.197, *p* < 0.01) which implied that heads of households usually know more about the plant. This is true since they handle home resource management and dietary choices. However, the findings also indicated a significant negative correlation between a respondent's age and their education level (*r* = −0.277, *p* < 0.01). This suggests a generational trend where older community members, who have lower formal education, are the primary holders of traditional knowledge. This is a critical finding, as this traditional knowledge is essential for recognizing and tending to the wild‐growing plant in its natural environment. Generally, the study revealed that both knowledge, attitude and practice scores were correlated with each other suggesting that knowledge is associated with more positive attitudes and better practices.

**TABLE 10:** Correlation coefficients between KAP of *J. heterocarpa* and socio‐ demographic characteristics.

Correlations	Sex	Division	HHZ	Age	ED	OC	HOH	Training	HB	Knowledge	Attitude	Practice
Sex	1											
Division	−0.039	1										
HHZ	−0.113	−0.125	1									
Age	−0.003	0.05	0.111	1								
ED	−0.022	0.071	0.099	−0.277**	1							
OC	−0.111	0.117	0.048	−0.251**	0.232**	1						
HOH	−0.242**	−0.066	0.055	−0.255**	0.049	−0.017	1					
Training	0.042	−0.054	−0.04	−0.159*	0.112	0.056	−0.004	1				
HB	−0.161*	0.132	0.023	−0.004	0.067	0.154*	−0.038	0.09	1			
Knowledge	−0.001	−0.143	0.037	−0.077	−0.06	−0.062	0.197**	−0.023	−0.625**	1		
Attitude	−0.122	0.002	−0.03	−0.118	−0.093	0.06	0.145	−0.026	−0.174*	0.390**	1	
Practice	0.009	−0.109	0.004	−0.003	−0.152*	−0.039	0.033	−0.1	−0.464**	0.535**	0.181*	1

Abbreviations: ED, education level; HB, health benefit; HHZ, house hold size; HOH, head of household; OC, occupation.

** Correlation is significant at the 0.01 level (2‐ tailed).

* Correlation is significant at the 0.05 level (2‐tailed).

## 4 Discussion

This study aims to evaluate the knowledge, attitudes, and behaviors (KAP) on the cultivation, post‐harvest management, processing, and consumption of *J. heterocarpa*, a prominent Indigenous vegetable among the Waluguru people residing in Kiroka Village, Morogoro Rural, Tanzania. The objective was to examine the community significance of this vegetable, its potential impact on food security, and the constraints associated with its utilization. The respondents' average ratings for knowledge, attitude, and behaviors were 48.6 ± 12.1, 51.4 ± 6.5, and 61.4 ± 6.7, respectively, indicating that the community possesses insufficient knowledge, a moderate attitude, and practices about the value chain of *J. heterocarpa*. Most respondents belonged to the young group between the ages of 29 and 39 years (Table 1). The significant percentage of people in this age category could be explained by most of them being women at child‐bearing age who are usually not formally employed, but run modest businesses close to their homes throughout the day which fits well with the interview time. Similar observations were reported by the study conducted by Mncwango et al. (2020). Additionally, more women were interviewed because they possessed specialized knowledge regarding the plant growth, post‐harvest handling and preparation methods of ILVs than men as highlighted by (Zulu et al. 2022). A low knowledge score indicates a deficient comprehension of multiple facets, including processing, nutritional composition, and preservation methods. Dlamini and Viljoen indicate that the decline in awareness of indigenous leafy vegetables (ILVs) as traditional dietary components is attributable to inadequate knowledge transmission between generations.

The moderate attitude score indicates that even with low knowledge of *J. heterocarpa* utilization, cultural values and dependence on traditional healers may contribute to a favorable community attitude towards its use. The study conducted by Kansiime et al. (2018) found contradictory findings where community members' negative views made it harder for them to consume most indigenous leafy vegetables (ILVs). Despite a knowledge gap, the moderate practice score indicated that the community used *J. heterocarpa* to some extent. The close relationship between *J. heterocarpa* and specific cultural ideas could explain its fair practices as it has been reported by Pawera et al. (2020) that, ILVs are well‐rooted in the cultural practices of a certain community. This study reported a significant proportion of respondents were ignorant of the health benefits associated with consuming *J. heterocarpa*. This could be the cause of low consumption and use of the plant as it has been reported by several researchers that lacking the information about the health benefits of ILVs causes low consumption rates (Chamara et al. 2021; Gido et al. 2017; Zulu et al. 2022).

The correlation between age and the decreasing presence of *J. heterocarpa* in its usual environment indicates a likely decline in its availability, jeopardizing its future use. The strong correlation between age and education, combined with the fact that younger generations do not recognize or tend to the plant, highlights a significant threat to the long‐term sustainability of *J. heterocarpa*. As the older generation ages, there is a risk that this vital traditional knowledge will be lost, which could lead to a decline in the wild plant population and its ultimate disappearance from the community's food system. The study conducted by Borelli et al. (2020) and Duguma (2020) underlined similar findings, emphasizing that the leading causes of the decline in wild edible plants were habitat degradation, development activity and land use changes.

The research indicated that knowledge ratings differed according to household size. Greater awareness of the utilization of *J. heterocarpa* was observed in larger households than in smaller ones. Gido et al. (2017) indicated analogous results, proposing that vegetable adoption is affected by household size, since bigger families experience greater ease in adoption due to a limited range of choices compared to smaller homes. The inadequate knowledge of storage and processing methods for *J. heterocarpa* caused notable post‐harvest losses during peak seasons. As shown in (Table 2), only 5.2% of respondents knew the best method to process the vegetable to increase its shelf life, and only 25.3% knew the correct post‐harvest handling practices. This demonstrates a significant knowledge gap that directly contributes to the challenges faced in the value chain. Other studies had similar results which indicated that most Indigenous leafy vegetables suffer around 50% post‐harvest losses (Elolu et al. 2023; Imathiu 2021; Kiringia 2019). About 47.7% of the respondents strongly agreed that the growing of *J. heterocarpa* benefited local farmers as shown in Table 4 (item A). Most of the Indigenous leafy vegetables are perceived as highly beneficial to local farmers due to their inherent low‐input traits, as they often grow wild and require minimal external resources or cultivation efforts. Other studies have reported similar findings that ILVs are important in rural farming systems (Chamara et al. 2021; Mungofa et al. 2022). However this statement contradicts the research showing that ILVs are well domesticated and marketed at more expensive rates since consumers are ready to pay upon knowing their nutritional value (Bokelmann et al. 2022; Imathiu 2021).

About 79.9% of respondents indicated that the activities associated with *J. heterocarpa* were predominantly occupied by women. This remark corresponds with the research carried out in Sri Lanka by Chamara et al. (2021), which shows that, in contrast to men, women participate mainly in all collecting, processing, and cooking activities of ILVs. These results contradict the study conducted in Mpumalanga, South Africa, where cultural standards forbid women in the reproductive cycle from engaging in ILVs or other agricultural activity (Zulu et al. 2022). Likewise, 52.3% of respondents “strongly disagreed” that *J. heterocarpa* was just a vegetable for the underprivileged and elderly, demonstrating tremendous high value for the vegetable which aligns with the findings of Chamara et al. (2021), which showed the community had a high positive attitude (98%) towards the utilization of ILVs. Conflicting research indicates that ILVs are regarded as outmoded food linked to underprivileged groups in society (Atuna et al. 2022; Irakoze et al. 2021; Nyonje et al. 2022). The study also found that perceptions towards *J. heterocarpa* were influenced by education. Respondents without formal education showed a higher mean attitude score. This suggested that the value of traditional knowledge and experiences, passed down from one generation to another, fosters a positive attitude within the community towards *J. heterocarpa* production and consumption. Traditional knowledge, often held by those with less formal education, may emphasize the cultural significance and practical uses of *J. heterocarpa*, leading to a strong appreciation for the plant. Similar findings have been reported by other researchers (Akinola et al. 2020; Bokelmann et al. 2022).

The study revealed a lack of seed storage for domestication practices. *J. heterocarpa* typically grows as a weed in agricultural settings, along roadsides, and in residential areas during the rainy season (Sangija, Martin, and Matemu 2022). Similar findings were reported in South Africa, where several indigenous leafy vegetables commonly grow alongside other crops in agricultural fields (Borelli et al. 2020; Mncwango et al. 2020). These findings align with the low domestication level of *J. heterocarpa* observed in the current study, which may be attributed to its widespread, uncultivated growth. However, this observation contrasts with studies that promote the use of certified seed systems for the commercialization of ILVs (Henze et al. 2020).

The study revealed modest consumption of *J. heterocarpa* whereas the majority of respondents consumed *J. heterocarpa* 2–3 times weekly which aligned with the findings of Chacha and Laswai (2020) on the consumption frequency of selected ILVs among respondents from Morogoro and Kilimanjaro Regions. However, the same studies have reported an increase in the consumption of ILVs in rural and peri‐urban areas (Atuna et al. 2022; Imathiu 2021). On the other hand, several studies have shown that ILVs consumption had declined compared to exotic vegetables (Borelli et al. 2020; Duguma 2020; Maseko et al. 2017). Those with enough food expenditures usually chose exotic veggies while eliminating ILVs from their diets (Atuna et al. 2022; Chacha and Laswai 2020). The study revealed that *J. heterocarpa* is primarily utilized in traditional ceremonies, such as weddings and puberty rites, where specific dishes are prepared to honor cultural heritage and preserve cultural identity. This finding is consistent with research showing that different cultural groups tend to consume particular ILVs, where their cooking and preparation techniques symbolize their cultural traditions (Chacha and Laswai 2020; Gido et al. 2017).

Practice scores were much influenced by age; the 18–28‐year age group showed a higher proportion of positive practice scores which suggested that younger respondents could follow better practices in the utilization of *J. heterocarpa* than the other age categories due to more exposure to schooling and modern farming methods. However other studies reported the contrary situation where younger individuals most of the time considered ILVs as out‐of‐date and connected to a lower socioeconomic class (Akinola et al. 2020; Imathiu 2021).

Along with *J. heterocarpa*, other ILVs existed and are consumed in diverse ways, which implies that the population has a range of foods to select from and is nutritionally secure. A study in Monduli, Tanzania, by Kansiime et al. (2018) reported a similar trend of preferred ILVs where the most preferred were pumpkin leaves followed by sweet potato leaves and nightshade while, *corchorus olitorius* was least accepted.

Food taboos and cultural restrictions around *J. heterocarpa* utilization suggested strongly rooted cultural views, social conventions, and traditional habits. Cultural limitations greatly limited the community's access to *J. heterocarpa*, therefore affecting the dietary diversity and nutritional availability. Nyonje et al. (2022) recorded comparable findings about Kenyan cultural attitudes about amaranth. The ban on the intake of ILVs for particular vulnerable populations, such as children and pregnant women, could be justified by their sensitivity to some substances, such as anti‐nutrients which could often hinder the absorption of essential minerals and protein (Ramulondi et al. 2021).

The study revealed that a knowledge score was strongly positively correlated with a practice score, meaning those who knew more about *J. heterocarpa* were more likely to make use of it. This corresponds with the broader idea of healthy behavior, whereby knowledge is usually a prerequisite for action (Bokelmann et al. 2022; Chamara et al. 2021). The results guided the intervention plan, cleared the relationship among the KAP variables, and pointed up the current disparity. Cultural barriers, such as the obstacle to *J. heterocarpa* consumption among some members of the community, could have affected the association between attitude and practices. The study carried out in Sri Lanka revealed a notable relationship between views on healthy eating and nutritional knowledge (Weerasekara et al. 2020). The cultural background of consumers influences the perception of food, which in turn affects their usage and acceptance of it (Jeong and Lee 2021).

The limited availability of *J. heterocarpa* in the dry season (93.1%) presents a considerable challenge for many respondents. A limited timeframe for harvesting exists, as the plant deteriorates rapidly in the absence of water. During the cold seasons especially June and July in Morogoro a notable period of dormancy occurs, marked by leaf thinning which suggests that the seasonal unavailability of the vegetable is a notable barrier, Several studies have reported similar observations that most of ILVs due to their wild nature, are scary during the dry seasons (Gido et al. 2017; Misci et al. 2021; Sangija, Kazosi, et al. 2022). Lack of awareness of the health benefits of *J. heterocarpa* in the community was a significant barrier to the utilization of this food.

Significant challenge hindering communities from fully utilizing ILVs like *J. heterocarpa* was the lack of processing and preservation methods that could enhance their shelf life. Reduced availability during dry seasons is likely due to increased post‐harvest losses from insufficient processing and preservation knowledge. Heaney et al. (2024), Henze et al. (2020) and Zulu et al. (2022) reported similar findings, which showed that training in food processing and preservation could enhance the market value and nutritional quality of locally grown vegetables, thus supporting local economies and household food security.

## 5 Conclusion and Recommendations

The study confirms that despite its cultural importance, the full potential of *J. heterocarpa* within the Waluguru community is not being realized. While the community shows a moderately positive attitude and demonstrates basic practices, a significant knowledge gap impedes its wider utilization. This knowledge deficiency, particularly concerning cultivation and post‐harvest handling, is a key driver of limited use and notable post‐harvest losses. The findings also reveal a critical disconnect: a generational knowledge gap where older members are the primary custodians of traditional wisdom, while younger generations are less engaged. Furthermore, negative socio‐cultural perceptions, particularly food taboos, remain a significant barrier to consumption.

In light of these findings, a comprehensive intervention is essential to enhance the plant's contribution to food security and nutrition. We recommend a culturally sensitive approach that leverages traditional knowledge and beliefs while introducing modern techniques for cultivation and processing. Such a strategy, by addressing knowledge deficits and negative perceptions directly, will empower the community to overcome these challenges and fully integrate *J. heterocarpa* into a sustainable and resilient food system.

### 5.1 Study Limitation

Given the exploratory nature of this study, the sample size was judged appropriate for an initial evaluation of knowledge, attitudes, and behaviors about the use of *J. heterocarpa* in Kiroka Ward. Further research involving more people is necessary to validate these findings.

The inclusion of double‐barreled questions in our survey may have affected the accuracy of our findings. Furthermore, although employing the chi‐square test on categorized Likert scale data is a standard approach, it may have led to a loss of certain nuances inherent in the ordinal data. Future research may employ advanced statistical methods to facilitate a more comprehensive analysis.

## Author Contributions

**Zenorina Aloyce Swai:** conceptualization (lead), data curation (lead), formal analysis (lead), funding acquisition (supporting), investigation (lead), methodology (lead), project administration (equal), resources (supporting), software (supporting), supervision (supporting), validation (equal), visualization (supporting), writing – original draft (lead), writing – review and editing (lead). **Frida Albinusi Nyamete:** conceptualization (supporting), data curation (supporting), formal analysis (supporting), funding acquisition (supporting), investigation (supporting), methodology (supporting), resources (supporting), software (supporting), supervision (lead), validation (supporting), visualization (supporting), writing – original draft (supporting), writing – review and editing (supporting). **Valerian C. K. Silayo:** conceptualization (supporting), data curation (supporting), formal analysis (supporting), funding acquisition (supporting), investigation (supporting), methodology (supporting), project administration (supporting), resources (supporting), software (supporting), supervision (lead), validation (supporting), visualization (supporting), writing – original draft (supporting), writing – review and editing (supporting).

## Conflicts of Interest

The authors declare no conflicts of interest.

## Data Availability

The raw data supporting the conclusions of this article is not publicly deposited due to the sensitive nature of the human participant information gathered through survey. However, upon reasonable request, the supplement material will be provided by the corresponding author under the ethical guidelines.
